# Choledocholithiases in a child with Hemoglobin Evans [alpha2 62(E11) Val→Met]

**DOI:** 10.1002/ccr3.588

**Published:** 2016-06-01

**Authors:** Itaru Hayakawa, Hiroshi Hataya, Takashi Kaneko

**Affiliations:** ^1^ Department of General PediatricsTokyo Metropolitan Children's Medical CenterTokyoJapan; ^2^ Department of Hematology and OncologyTokyo Metropolitan Children's Medical CenterTokyoJapan

**Keywords:** Choledocholithiasis, Hemoglobin Evans [alpha2 62(E11) Val→Met], hemolytic anemia, unstable hemoglobin

## Abstract

We present the first description of choledocholithiases in a 10‐year‐old boy with Hemoglobin Evans. Although biliary stones are much less common in children than in adults, epigastric pain in children with known hemolytic status should alert the physician to the possibility of biliary colic.

## Case Presentation

Ten‐year‐old Japanese boy with past medical history of Hemoglobin Evans [alpha2 62(E11) Val→Met] and asymptomatic gallbladder stones was admitted for epigastric abdominal pain. He had reported nothing out of the ordinary until the day before admission, when he experienced acute‐onset abdominal pain and emesis. The pain subsided spontaneously after several hours, but recurred soon after breakfast the next morning. The pain was continuously located in the epigastric region. The patient described the pain as 8 on a scale of 0–10, with 10 indicating the severest pain. A review of systems showed the patient positive for general malaise, nonbiliary vomiting, and conjunctiva which are slightly more yellowish than his usual state. There was no fever, back pain, altered mental status, or chest pain.

His past medical history was significant for a heterozygous in‐frame mutation of Hemoglobin Evans [alpha2 62(E11) Val→Met]. He had been followed up by a pediatric hematologist for resultant mild hemolytic anemia and indirect‐bilirubin‐dominant jaundice (total bilirubin 4–6 mg/dL), but was otherwise healthy with normal motor and cognitive development. When he was 8‐years old, aplastic crisis due to parvovirus B19 infection occurred. At that time, an abdominal ultrasonography revealed asymptomatic gallbladder stones. He took daily folic acid and ursodeoxycholic acid. Both his mother and his brother carried the same heterozygous mutation.

On examination, the patient was not in acute distress. His height was 133 cm and his weight was 30.5 kg. He was afebrile, his heart rate was 62 beats per minute, his respiratory rate was 23 breaths per minute, and his extremities were warm. The skin and conjunctiva were icteric. The abdomen was soft and flat without guarding. He reported epigastric tenderness on palpation. The liver and spleen were not palpable. Murphy's sign was negative.

Laboratory studies revealed marked hyperbillirubinemia and elevated liver and biliary function, with T‐Bil of 13.9 mg/dL, D‐Bil of 4.5 mg/dL, AST 371 U/L, ALT 438 U/L, LDH 419 U/L, ALP 929 U/L, and g‐GT 71 U/L. There was no leukocytosis (WBC 7950/μL), or any elevation of serum amylase (Amy 58 U/L). Normocytic anemia and reticulocytosis (Hemoglobin 10.9 g/dL, MCV 95.6 fL, reticulocyte 12.7%) were unchanged from his previous visit. An abdominal ultrasonography and contrast‐enhanced computed tomography (CT) scan were conducted in order to determine the cause of the obstructive jaundice. Ultrasonography revealed a dilated common bile duct (3.6 mm in diameter), but failed to reveal the cause of the obstruction. The CT scan showed multiple, radiopaque shadows in the lower common bile duct as well as in the gallbladder. The pancreatic duct was not dilated.

The diagnosis of choledocholithiases and obstructive jaundice was made. Once oral intake was stopped, the abdominal tenderness disappeared quickly. The jaundice worsened, however, over the next 4 days and reached T‐bil/D‐Bil of 25.6/14.2 mg/dL, respectively. A refractory, itchy irritation of the skin developed. We performed endoscopic retrograde cholangiopancreatography (ERCP) on 6th day of hospitalization. Endoscopic papillary balloon dilatation, followed by stone removal procedure using a basket and an extractor balloon resulted in extraction of a small bilirubin stone to the duodenum. Although cholangiography after the stone removal revealed no definite residual stones, a biliary stent (Flexima^™^ biliary stent system, 7 Fr, 5 cm; Boston Scientific Japan, Tokyo, Japan) was placed in order to prevent recurrence of symptoms. Jaundice and general malaise subsided after the procedure. A laparoscopic cholecystectomy and intraoperative cholangiography were performed 10 days after the ERCP. Intraoperative cholangiography revealed a new choledocholithiasis adjacent to the patent biliary stent placed during the first ERCP. Attempts to remove the stone failed. The gallbladder contained many black pigment stones of several millimeters in size. He was discharged without symptoms.

The remaining bilirubin choledocholithiasis was removed during the second ERCP, performed 4 months after the cholecystectomy. Although cholangiography after stone retrieval showed no evidence of remaining biliary stones, a biliary stent was kept in place for 2 more months to prevent any possible biliary obstruction, and was finally removed 2 months after the second ERCP. The symptoms have not recurred to date.

## Discussion

We experienced a 10‐year‐old boy with a history of Hemoglobin Evans and accompanying hemolytic anemia who suffered worsening obstructive jaundice due to choledocholithiases. He was successfully treated with an ERCP and cholecystectomy. His past medical history and postprandial pain exacerbation significantly facilitated early diagnosis and management.

Hemoglobin Evans is a rare, inherited, unstable hemoglobin variant 1, 2. Only several lineages have ever been reported worldwide 1, 2, 3, 4. Patients who are heterozygous for this rare in‐frame mutation suffer several complications, including mild hemolytic anemia, splenomegaly, and aplastic crisis 4, but biliary stones have hitherto never been reported. Here, we describe the first case of choledocholithiases requiring intervention in a child presenting Hemoglobin Evans.

Choledocholithiases, as well as cholelithiases, are much rarer in children than in adults 5. Children with cholelithiasis reportedly tend to possess some underlying diseases which accelerate biliary stone formation 6. Thus, children with choledocholithiases should be examined for an underlying cause of the biliary stones, such as hemolytic anemia, dyslipidemia, congenital biliary dilatation, and so on.

Hemoglobin Evans is one of the unstable hemoglobin variants. Unstable hemoglobins decrease their solubility in red cells. As a result of precipitating hemoglobins, red cells carrying unstable hemoglobins have decreased life spans. Destruction and overproduction of red cells then leads to hemolytic anemia and pigment stone formation of varying severity. It is reported that Hemoglobin Evans often leads to mild hemolytic anemia 3, but the incidence of biliary stone formation due to this disease is not known. The present report is the first of choledocholithiases in a patient with Hemoglobin Evans. Our case illustrates the fact that those with chronic hemolytic anemia are at high risk of developing black pigment bilirubin stones. Thus, when children with hemolytic anemia present with abdominal pain, the possibility of cholecystitis or cholangitis should always be kept in mind, since cholelithiasis is not rare in this population 7.

Drainage of the bile and removal of the stone are the two most important aspects of the treatment of choledocholithiases 8, 9. Recently, ERCP has emerged as a useful method of both drainage and stone removal in pediatric patients with choledocholithiases 10. In our case, the first ERCP resulted in successful drainage, but the choledocholithiasis was evident on intraoperative cholangiography 2 weeks after the first ERCP. Since obstructive jaundice had resolved by the time the cholangiography was conducted, a second, elective round of ERCP was performed 4 months later, successfully removing the residual stone. As in our case, the choledocholithiases frequently require both surgical and endoscopic intervention 8, 9.

Since hemolytic anemia is not improved by a cholecystectomy, our patient is at risk of a recurrence of the choledocholithiases. Indeed, 19.6% of patients with sickle cell disease who received a cholecystectomy suffered recurrent biliary tract diseases after the procedure 11. Although the prevalence of recurrent choledocholithiases in patients with unstable hemoglobin variants is unknown, our patient requires continued monitoring against the potential recurrence choledocholithiases.

In this report, we described for the first time ever a case of choledocholithiases in a child with Hemoglobin Evans. Although biliary stones are less common in children than in adults, children with hemolytic status are at high risk of developing bilirubin stones. Thus, when children with known hemolytic status report epigastric pain, the possibility of choledocholithiases should be considered, and detailed history taking, physical examinations, as well as laboratory and radiological testing, are warranted.

## Conflict of Interest

None declared.
